# Sensitivity analyses gain relevance by fixing parameters observable during the empirical analyses

**DOI:** 10.1002/gepi.22530

**Published:** 2023-07-07

**Authors:** Gibran Hemani, Apostolos Gkatzionis, Kate Tilling, George Davey Smith

**Affiliations:** 1 MRC Integrative Epidemiology Unit, University of Bristol

Dear Editor

In 2017 we presented the MR Steiger method for inferring causal directions between variables^1^. We discussed many of its potential limitations including that unmeasured confounding under certain extreme circumstances could lead to the wrong inferred causal direction. Lutz et al (2022)^2^ propose an R package (UCRMS) for performing sensitivity analysis of the MR Steiger method, and use it in an illustration to suggest that the MR Steiger method has a ~90% chance of giving the wrong answer due to unmeasured confounding. In this note we will show that an error in their approach to sensitivity analysis leads to the wrong conclusion about the validity of the MR Steiger test. We provide a valid alternative which uses the observed data to investigate sensitivity to unmeasured confounding.

A sensitivity analysis aims to understand the degree to which a result can change due to uncertainties in the inputs^3^. In this case for the MR Steiger test, we need to ask how sensitive is the inference of the causal direction between X and Y to possible values of unmeasured confounders influencing X and Y. Importantly, there is relative certainty in many of the parameters of this system because they are easily observed, for example, the variances of X, Y and the instruments, the estimated effect of the instruments on X and Y, and therefore the instrumental variable estimate of the effect of X on Y. Often the ordinary least squares (OLS) association between X and Y is also available either due to the analysis being performed using individual level data, or by sourcing the estimate from other published results. Therefore, an appropriate sensitivity analysis must explore the extent to which the inferred causal direction between X and Y can change due to unmeasured confounding, without causing these observed parameters to change.

Lutz et al’s proposed method does not attempt to fix all observable parameters. In the simple example provided by Lutz et al the variance of Y varies between 28 and 39, and the OLS estimate varies between 1 and -1 across the parameter values used for the sensitivity analysis. This arises because the residual variance – which is fixed in their approach -, is unobserved. Instead the phenotypic variance - which is observed – should be fixed. If they were presenting a simulation of the general performance of MR Steiger under unmeasured confounding then it would not matter that the simulated parameters are not tied to those observed in a particular empirical analysis. However in a sensitivity analysis, allowing observed parameters to vary provides no value to the analyst. To say that unmeasured confounding could reverse the causal direction, provided that the variance of Y also changes drastically, is of little use to the researcher who has a dataset with an observed variance of Y. If some quantities are observed (i.e. the regression coefficient for Y on X, the variance explained in X by the instrument, the variances of X and Y, and the IV effect estimate are all observed), allowing only *β_uy_* and *β_ux_* to vary and compensating through changing the residual variance, the SNP-outcome *R*^2^ will not change under any set of *β_uy_* and *β_ux_* parameters (Supplementary Note).

Briefly expanding on Lutz et al’s analysis, they specified that for a causal effect of *β_xy_* = 1, there were specific unmeasured confounding parameters of *β_ux_* = –5 and *β_uy_* ranged only between 0 and 11. Using these parameters they suggest that the MR Steiger method has a ~90% chance of returning the incorrect causal direction. But if *β_ux_* and *β_uy_* were permitted the same ranges of values (e.g. -11 to 11) then the Steiger method would only return the incorrect result in 36% of confounding scenarios. If the range was restricted to -1 to 1 for *β_ux_* and *β_uy_* each, then the wrong result would only be found in 0.02% of scenarios. In our 2017 paper (Supplementary note 3) we analysed a much broader range of scenarios to comprehensively assess the degree to which unmeasured confounding could in general introduce a problem, and concluded that in most practical cases, where $R_{xy}^{2}<0.2$, the chance of unmeasured confounding leading to the incorrect causal direction was very small.

If analysts were motivated to check the sensitivity of MR Steiger to unmeasured confounding, a different approach is needed where one asks what values of unmeasured confounding support the inferred causal direction for a given set of empirically observed quantities (variances of X, Y and instrument, effects of instrument on X and Y, and OLS estimate of X on Y). Analysts can then determine whether the confounding values required to cast doubt their conclusion are plausible. Alternatively one can determine what fraction of the possible confounding parameter space supports the inferred causal direction. In the Supplementary Note we provide an analytical solution to this problem. We illustrate that at the analysis-specific level the probability that unmeasured confounding will reverse the causal direction inferred by MR Steiger only exceeds a low probability when confounders explain large fractions of the variance in X and Y. The method is included in the TwoSampleMR package and is fast because it uses a closed form calculation rather than the stochastic simulation approach implemented by UCRMS.

## Supplementary Material

Supplementary note

## Data Availability

All code used to perform the analysis is available at https://github.com/explodecomputer/steigercollider/.
