# Supplementary material for: Sensitivity analyses gain relevance by fixing parameters observable during the empirical analyses
Source: Genet Epidemiol. Author manuscript; Available in PMC 2024 Dec 24. (PMC7617259; doi:10.1002/gepi.22530)
Supplement: Supplementary note [file EMS184864-supplement-Supplementary_note.pdf]

Supplementary note - Unmeasured confounding

Gibran Hemani, Kate Tilling, George Davey Smith

Background

The MR Steiger method uses instrumental variables to infer the causal orientation between two variables. Once an orientation is inferred there is a question as to whether the inference is erroneous due to processes that can bias the MR Steiger method. Here we focus on unmeasured confounding. Suppose that X is inferred to be causal for Y, we ask whether some combination of confounding parameters could give rise to the inferred direction under a data generating model in which the reverse causal direction is true. Figure 1 illustrates the two competing causal directions.

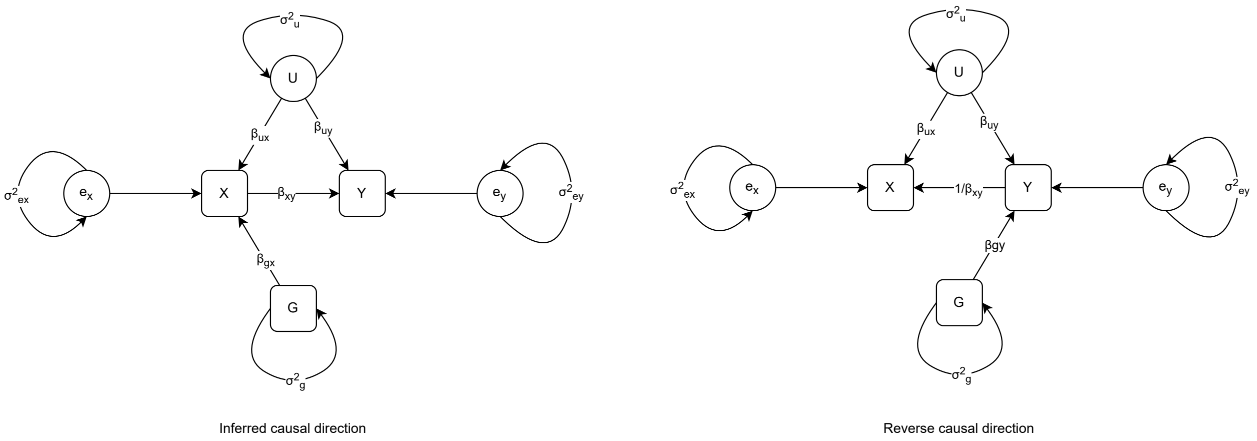

Figure 1: Two competing causal directions and their parameters represented as path diagrams.

According to the Inferred causal direction in figure 1,

$$X = \alpha_x + \beta_{gx} G + \beta_{ux} U + e_x$$

where SNP with allele frequency p has variance $\sigma^2_G = 2p(1-p)$, $U \sim N(0, \sigma^2_u)$ is an unmeasured confounder, and $e_x \sim N(0, \sigma^2_{e_x})$ is an error term. The variance of X will be

$$\sigma^2_x = \beta^2_{gx}\sigma^2_g + \beta^2_{ux}\sigma^2_u + \sigma^2_{e_x}$$

We can also write

$$Y = \alpha_y + \beta_{xy} X + \beta_{uy} U + e_y$$

where $e_y \sim N(0, \sigma^2_{e_y})$ is an error term. Going forwards intercept terms can be ignored. The variance of Y will be

$$\sigma^2_y = \beta^2_{xy}\beta^2_{gx}\sigma^2_g + \sigma^2_u(\beta_{xy}\beta_{ux} + \beta_{uy})^2 + \beta^2_{xy}\sigma^2_{e_x} + \sigma^2_{e_y}$$

The variance explained in X by G will be

$$R^2_{gx} = \frac{\beta^2_{gx}\sigma^2_g}{\beta^2_{gx}\sigma^2_g + \beta^2_{ux}\sigma^2_u + \sigma^2_{e_x}}$$

The variance explained in Y by G will be

$$R^2_{gy} = \frac{\beta^2_{gx}\beta^2_{xy}\sigma^2_g}{\beta^2_{xy}\beta^2_{gx}\sigma^2_g + \sigma^2_u(\beta_{xy}\beta_{ux} + \beta_{uy})^2 + \beta^2_{xy}\sigma^2_{e_x} + \sigma^2_{e_y}}$$

The variance explained in X by U will be

$$R^2_{ux} = \frac{\beta^2_{ux}\sigma^2_u}{\beta^2_{gx}\sigma^2_g + \beta^2_{ux}\sigma^2_u + \sigma^2_{e_x}}$$

The variance explained in Y by U will be

$$R^2_{uy} = \frac{\sigma^2_u(\beta_{uy} + \beta_{ux}\beta_{xy})^2}{\beta^2_{xy}\beta^2_{gx}\sigma^2_g + \sigma^2_u(\beta_{xy}\beta_{ux} + \beta_{uy})^2 + \beta^2_{xy}\sigma^2_{e_x} + \sigma^2_{e_y}}$$

Note that at this stage the asymptotic result of the computationally intensive simulations employed by Lutz et al could be derived analytically by observing that

$$\begin{aligned} R^2_{gx} > R^2_{gy} &\Rightarrow \frac{\beta^2_{gx}\sigma^2_g}{\beta^2_{gx}\sigma^2_g + \beta^2_{ux}\sigma^2_u + \sigma^2_{e_x}} > \frac{\beta^2_{gx}\beta^2_{xy}\sigma^2_g}{\beta^2_{xy}\beta^2_{gx}\sigma^2_g + \sigma^2_u(\beta_{xy}\beta_{ux} + \beta_{uy})^2 + \beta^2_{xy}\sigma^2_{e_x} + \sigma^2_{e_y}} \\ &\Rightarrow \beta^2_{uy}\sigma^2_u + 2\beta_{ux}\beta_{uy}\beta_{xy}\sigma^2_u + \sigma^2_{e_y} > 0 \end{aligned}$$

is a second-degree polynomial inequality for β_{uy} which can be solved as

$$\begin{aligned} \Delta &= 4\beta^2_{ux}\beta^2_{xy}\sigma^4_u - 4\sigma^2_u\sigma^2_{e_y} \\ &= 4\sigma^2_u(\beta^2_{ux}\beta^2_{xy} - \sigma^2_{e_y}) \end{aligned}$$

This means that the comparison of the two R^2 values reduces to the sign of the quantity $\beta^2_{ux}\beta^2_{xy} - \sigma^2_{e_y}$. If the latter is negative, $R^2_{gx} > R^2_{gy}$ holds regardless of the value of β_{uy} (and ignoring finite-sample variation). If it's positive, we will have $R^2_{gx} < R^2_{gy}$ in the interval $[\beta_1, \beta_2]$ where

$$\begin{aligned} \beta_{1,2} &= \frac{-2\beta_{xu}\beta_{xy}\sigma^2_u \pm \sqrt{\Delta}}{2\sigma^2_u} \\ &= \beta_{xu}\beta_{xy} \pm \sqrt{\beta^2_{xu}\beta^2_{xy} - \frac{\sigma^2_{e_y}}{\sigma^2_u}} \end{aligned}$$

As an application, for the parameter values used by Lutz et al. we obtain the reverse causal direction for β_{uy} values in the interval $[5 - 2\sqrt{6}, 5 + 2\sqrt{6}] \approx [0.1, 9.9]$.

However as described in the main text this does not serve as a sufficient sensitivity analysis because it allows observed quantitites to vary considerably. We continue now to derive a more appropriate sensitivity analysis that does fix observed quantitites.

In practice we tend to observe the following quantitites: β_{gx}, σ^2_g, σ^2_x, σ^2_y. The analysis is used to estimate β_{xy}. We can often obtain estimates of β_{OLS}. We do not know σ^2_u, β_{ux} or β_{uy}, but given estimates of β_{OLS}

and β_{xy} we can obtain possible values for these confounder parameters. The observational association in this system will be

$$\beta_{OLS} = \frac{\beta_{gx}^2 \beta_{xy} \sigma_g^2 + \beta_{ux}^2 \beta_{xy} \sigma_u^2 + \beta_{ux} \beta_{uy} \sigma_u^2 + \beta_{xy} \sigma_{e_x}^2}{\sigma_g^2 \beta_{gx}^2 + \sigma_u^2 \beta_{ux}^2 + \sigma_{e_x}^2}$$

Hence the association between X and Y due to confounding will be

$$\begin{aligned} \beta_C &= \beta_{OLS} - \beta_{xy} \\ &= \frac{\beta_{ux} \beta_{uy} \sigma_u^2}{\sigma_g^2 \beta_{gx}^2 + \sigma_u^2 \beta_{ux}^2 + \sigma_{e_x}^2} \end{aligned}$$

The key question is this: **If β_{gx}, σ_g^2, σ_x^2, σ_y^2, $\hat{\beta}_{OLS}$ and $\hat{\beta}_{xy}$ are fixed, are there values of R_{ux} and R_{uy} that can satisfy either X being causal for Y or Y being causal for X?** We approach this question by analytically exploring this possible confounding parameter space. The possible range of U-X confounding is

$$\beta_{ux} \in \left\{ -\sqrt{\frac{\sigma_x^2 - \beta_{gx}^2 \sigma_g^2}{\sigma_u^2}}, \sqrt{\frac{\sigma_x^2 - \beta_{gx}^2 \sigma_g^2}{\sigma_u^2}} \right\}$$

which means that for any particular value of β_{ux} within this range the values of

$$\sigma_{e_x}^2 = \sigma_x^2 - \beta_{gx}^2 \sigma_g^2 - \beta_{ux}^2 \sigma_u^2$$

and

$$\beta_{uy} = \beta_C \frac{\beta_{gx}^2 \sigma_g^2 + \beta_{ux}^2 \sigma_u^2 + \sigma_{e_x}^2}{\beta_{ux} \sigma_u^2}$$

and

$$\sigma_{e_y}^2 = \sigma_y^2 - \beta_{xy}^2 \beta_{gx}^2 \sigma_g^2 - \sigma_u^2 (\beta_{xy} \beta_{ux} + \beta_{uy})^2 - \beta_{xy}^2 \sigma_{e_x}^2$$

can be inferred directly once the value of σ_u^2 is set to 1 for convenience. Overall, through this set of equations, we can obtain confounding values that could give rise to the observed quantitites under either the inferred causal direction or the reverse causal direction. In the case of the reverse causal direction the value of $\beta_{xy,rev} = 1/\beta_{xy}$ and $\beta_{OLS,rev} = \beta_{OLS} \sigma_x^2 / \sigma_y^2$, $\beta_{gx,rev} = \beta_{gx} \beta_{xy}$. The sensitivity analysis proceeds by identifying the values of R_{ux}^2 and R_{uy}^2 that agree or disagree with the inferred causal direction, allowing the analyst to make a judgement as to the reliability of the inference. Building on this, one can determine the probability of the inferred causal direction being correct by framing it in a Bayesian context. Consider the value C being 1 if the values of R_{ux}^2 and R_{uy}^2 agree with the inferred causal direction, and 0 otherwise. We implement the method using a beta-distributed prior for confounding values with shape parameters a and b for reasons described later, though analysts could specify alternative prior distributions. Across all possible values of R_{ux}^2 and R_{uy}^2 the probability that the inferred direction is correct is given by

$$p(C|a, b) = \int p(C|R_{ux}^2, R_{uy}^2) p(R_{ux}^2, R_{uy}^2|a, b)$$

A posterior probability close to 1 will suggest that there is relatively little chance of the inferred direction being incorrect due to unmeasured confounding. Choosing shape parameters of $a = 1$ and $b = 1$ would give

a flat prior, where all confounding values are equally likely. Elevating b to a larger value will downweight very large confounding values, while reducing a to a lower value will enforce more weight for confounding close to 0. To be conservative we would recommend using a flat prior, whereas values of $a = 1, b = 10$ might be more realistic which eliminates unrealistically large confounding values. Note that for simplicity, our implementation doesn't calculate the integral function directly, rather for a dense range of R^2_{ux} and R^2_{uy} values we find the fraction of instances in which the inferred causal direction is supported, weighting the contribution of each parameter value by the weight associated with the specified prior distribution.

Note also that if the OLS estimate is unknown then analysts can specify a range of plausible values to be evaluated.

Analysis

```
library(dplyr)
library(ggplot2)
library(latex2exp)
library(ieugwasr)
library(TwoSampleMR)
```

This function obtains the rsq values given fixed parameters

```
get_calcs <- function(bxy, bgx, bux, buy, vg, vu, vex, vey) {
    args <- as.list(environment())
    bxyo <- ((bgx^2*bxy*vg + bux^2*bxy*vu + bux*buy*vu + bxy*vex) / (vg*bgx^2 + vu*bux^2 + vex))
    vx <- bgx^2 * vg + bux^2 * vu + vex
    vy <- bxy^2*bgx^2*vg + (bxy*bux+buy)^2*vu + bxy^2*(vex) + vey
    conf <- bux * vu * buy / (vg * bgx^2 + vu * bux^2 + vex)
    rsqxyo <- bxyo^2 * vx / vy
    rsqxyos <- rsqxyo * sign(bxyo)
    rsqxy <- bxy^2 * vx / vy
    rsqxys <- rsqxy * sign(bxy)
    rsqgx <- bgx^2*vg / (bgx^2 * vg + bux^2 * vu + vex)
    rsqgy <- bgx^2*bxy^2*vg / (bxy^2*bgx^2*vg + (bxy*bux+buy)^2*vu + bxy^2*(vex) + vey)
    rsqux <- bux^2*vu / (bgx^2 * vg + bux^2 * vu + vex)
    rsquy <- (buy + bux * bxy)^2 * vu / (bxy^2*bgx^2*vg + (bxy*bux+buy)^2*vu + bxy^2*(vex) + vey)
    rsquxs <- rsqux * sign(bux)
    rsquys <- rsquy * sign(buy)
    return(c(args, list(
        vx=vx,
        vy=vy,
        bxyo=bxyo,
        conf=conf,
        rsqgx=rsqgx,
        rsqgy=rsqgy,
        rsqux=rsqux,
        rsquy=rsquy,
        rsqxy=rsqxy,
        rsqxyo=rsqxyo,
        rsqxyos=rsqxyos,
        rsquxs=rsquxs,
        rsquys=rsquys,
        rsqxys=rsqxys
    )))
}
```

Check by comparing to simulated individual level data

```
get_calcs_id <- function(bxy, bgx, bux, buy, vg, vu, vex, vey, n=500000){
    u <- rnorm(n, sd = sqrt(vu))
    g <- rnorm(n, sd = sqrt(vg))
    ex <- rnorm(n, sd = sqrt(vex))
    ey <- rnorm(n, sd = sqrt(vey))
    x <- u * bux + g * bgx + ex
    y <- u * buy + x * bxy + ey
    res <- tibble(
        bxyo=cov(x,y)/var(x),
        rsqux=cor(u,x)^2,
        rsquy=cor(u,y)^2,
        rsqxyo=cor(x,y)^2,
        rsqxyos=rsqxyo*sign(bxyo),
        rsquxs=rsqux*sign(bux),
        rsquys=rsquy*sign(buy),
        rsqgx = cor(g,x)^2,
        rsqgy = cor(g,y)^2
    )
    return(res)
}
bind_rows(get_calcs(0.1, 1, 1, 1, 0.5, 0.1, 0.4, 0.9-0.1^2) %>% as_tibble(),
get_calcs_id(0.1, 1, 1, 1, 0.5, 0.1, 0.4, 0.9-0.1^2) %>% as_tibble())
```

```
## # A tibble: 2 x 22
##     bxy   bgx   bux   buy    vg    vu   vex   vey    vx    vy  bxyo  conf rsqgx
##   <dbl> <dbl> <dbl> <dbl> <dbl> <dbl> <dbl> <dbl> <dbl> <dbl> <dbl> <dbl> <dbl>
## 1   0.1     1     1     1   0.5   0.1   0.4  0.89     1  1.02 0.2     0.1 0.5
## 2  NA      NA    NA    NA  NA    NA    NA    NA      NA NA    0.200  NA   0.498
## # ... with 9 more variables: rsqgy <dbl>, rsqux <dbl>, rsquy <dbl>,
## #   rsqxy <dbl>, rsqxyo <dbl>, rsqxyos <dbl>, rsquxs <dbl>, rsquys <dbl>,
## #   rsqxys <dbl>
```

Parameter ranges used by Lutz et al

```
get_calcs(1, 1, -5, seq(0,11,by=1), 1, 1, 1, 1) %>% as_tibble() %>% dplyr::select(bux, buy, vx, vy, rsqg
```

```
## # A tibble: 12 x 6
##      bux   buy    vx    vy  rsqgx  rsqgy
##    <dbl> <dbl> <dbl> <dbl>  <dbl>  <dbl>
##  1    -5     0    27    28 0.0370 0.0357
##  2    -5     1    27    19 0.0370 0.0526
##  3    -5     2    27    12 0.0370 0.0833
##  4    -5     3    27     7 0.0370 0.143
##  5    -5     4    27     4 0.0370 0.25
##  6    -5     5    27     3 0.0370 0.333
##  7    -5     6    27     4 0.0370 0.25
##  8    -5     7    27     7 0.0370 0.143
##  9    -5     8    27    12 0.0370 0.0833
## 10    -5     9    27    19 0.0370 0.0526
## 11    -5    10    27    28 0.0370 0.0357
## 12    -5    11    27    39 0.0370 0.0256
```

Calculate the values of rux and ruy that would satisfy the fixed parameters

```
sens <- function(bxy=0.1, bxyo=0.2, bgx=0.5, vx=1, vy=1, vu=1, vg=0.5, simsize=100) {
    # vx <- bgx^2 * vg + p$bux_vec^2 * vu + vex
    bux_lim <- sqrt((vx - bgx^2 * vg)/vu)
    bux_vec <- seq(-bux_lim, bux_lim, length.out=simsize)
    # Allow causal effect to vary by +/- 200%
    vex <- vx - bgx^2 * vg - bux_vec^2 * vu
    conf <- bxyo - bxy
    buy_vec <- conf * (bgx^2*vg + bux_vec^2*vu + vex) / (bux_vec * vu)
    vey <- vy - (bxy^2*bgx^2*vg + (bxy*bux_vec+buy_vec)^2*vu + bxy^2*vex)
    # vy <- bxy^2*bgx^2*vg + (bxy*bux_vec+buy_vec)^2*vu + bxy^2*vex + vey
    bux_vec * vu * buy_vec / (vg * bgx^2 + vu * bux_vec^2 + vex)
    res <- get_calcs(bxy, bgx, bux_vec, buy_vec, vg, vu, vex, vey) %>%
      as_tibble() %>%
      mutate(bxy=bxy, bgx=bgx, bux=bux_vec, buy=buy_vec, vg=vg, vu=vu, vex=vex, vey=vey)
    return(res)
}
```

Test sensitivity analysis

```
u_sensitivity <- function(bxy, bxyo, bgx, vx, vy, vg, vu = 1, simsize=10000, beta_a=1, beta_b=1, plot=T
{
    o <- params <- bind_rows(
        sens(bxy=bxy, bxyo=bxyo, bgx=bgx, vx=vx, vy=vy, vu=vu, vg=vg, simsize=simsize) %>%
            mutate(direction="inferred"),
        sens(bxy=1/bxy, bxyo=bxyo * vx / vy, bgx=bgx * bxy, vx=vy, vy=vx, vu=vu, vg=vg, simsize=simsize
            mutate(direction="reverse")
    ) %>%
    filter(
        vex >= 0 &
        vey >= 0 &
        rsquy >= 0 & rsquy <= 1 &
        rsqux >= 0 & rsqux <= 1 &
        rsqgx >= 0 & rsqgx <= 1 &
        rsqgy >= 0 & rsqgy <= 1
    ) %>%
        group_by(direction) %>%
        do({
            x <- .
            x1 <- x$rsqux[-1]
            x2 <- x$rsqux[-length(x$rsqux)]
            y1 <- x$rsquy[-1]
            y2 <- x$rsquy[-length(x$rsquy)]
            d <- sqrt((x1-x2)^2 + (y1-y2)^2)
            d[d > quantile(d, na.rm=T, probs=0.99)*4] <- NA
            x$d <- c(NA, d)
            x$weight <- dbeta(x$rsqux, shape1=beta_a, shape2=beta_b) * dbeta(x$rsquy, shape1=beta_a, sha
            x
        })

    w <- o$d * o$weight
    w1 <- w[o$direction=="inferred"]
    prop <- sum(w1, na.rm=T) / sum(w, na.rm=T)
    ret <- list(result=o, prop=prop)
    if(plot) {
```

```
        ret$pl <- ggplot(o, aes(x=rsquxs, y=rsquys)) +
        geom_point(aes(colour=direction, size=weight))
    }
    return(ret)
}
```

Example 1 - bxy and bxyo are similar, and confounders that explain more of the variance are strongly downweighted

```
r <- u_sensitivity(bxy=0.1, bxyo=0.2, bgx=0.5, vx=1, vy=1, vg=0.5, beta_a=1, beta_b=10)
r
```

```
## $result
## # A tibble: 8,958 x 25
## # Groups:   direction [2]
##      bxy   bgx    bux    buy    vg    vu      vex   vey    vx    vy  bxyo  conf
##    <dbl> <dbl>  <dbl>  <dbl> <dbl> <dbl>    <dbl> <dbl> <dbl> <dbl> <dbl> <dbl>
##  1   0.1   0.5 -0.935 -0.107   0.5     1 0        0.959     1     1   0.2   0.1
##  2   0.1   0.5 -0.935 -0.107   0.5     1 0.000350 0.959     1     1   0.2   0.1
##  3   0.1   0.5 -0.935 -0.107   0.5     1 0.000700 0.959     1     1   0.2   0.1
##  4   0.1   0.5 -0.935 -0.107   0.5     1 0.00105  0.959     1     1   0.2   0.1
##  5   0.1   0.5 -0.935 -0.107   0.5     1 0.00140  0.959     1     1   0.2   0.1
##  6   0.1   0.5 -0.934 -0.107   0.5     1 0.00175  0.959     1     1   0.2   0.1
##  7   0.1   0.5 -0.934 -0.107   0.5     1 0.00210  0.959     1     1   0.2   0.1
##  8   0.1   0.5 -0.934 -0.107   0.5     1 0.00245  0.959     1     1   0.2   0.1
##  9   0.1   0.5 -0.934 -0.107   0.5     1 0.00280  0.959     1     1   0.2   0.1
## 10   0.1   0.5 -0.934 -0.107   0.5     1 0.00315  0.959     1     1   0.2   0.1
## # ... with 8,948 more rows, and 13 more variables: rsqgx <dbl>, rsqgy <dbl>,
## #   rsqux <dbl>, rsquy <dbl>, rsqxy <dbl>, rsqxyo <dbl>, rsqxyos <dbl>,
## #   rsquxs <dbl>, rsquys <dbl>, rsqxys <dbl>, direction <chr>, d <dbl>,
## #   weight <dbl>
##
## $prop
## [1] 1
##
## $pl
```

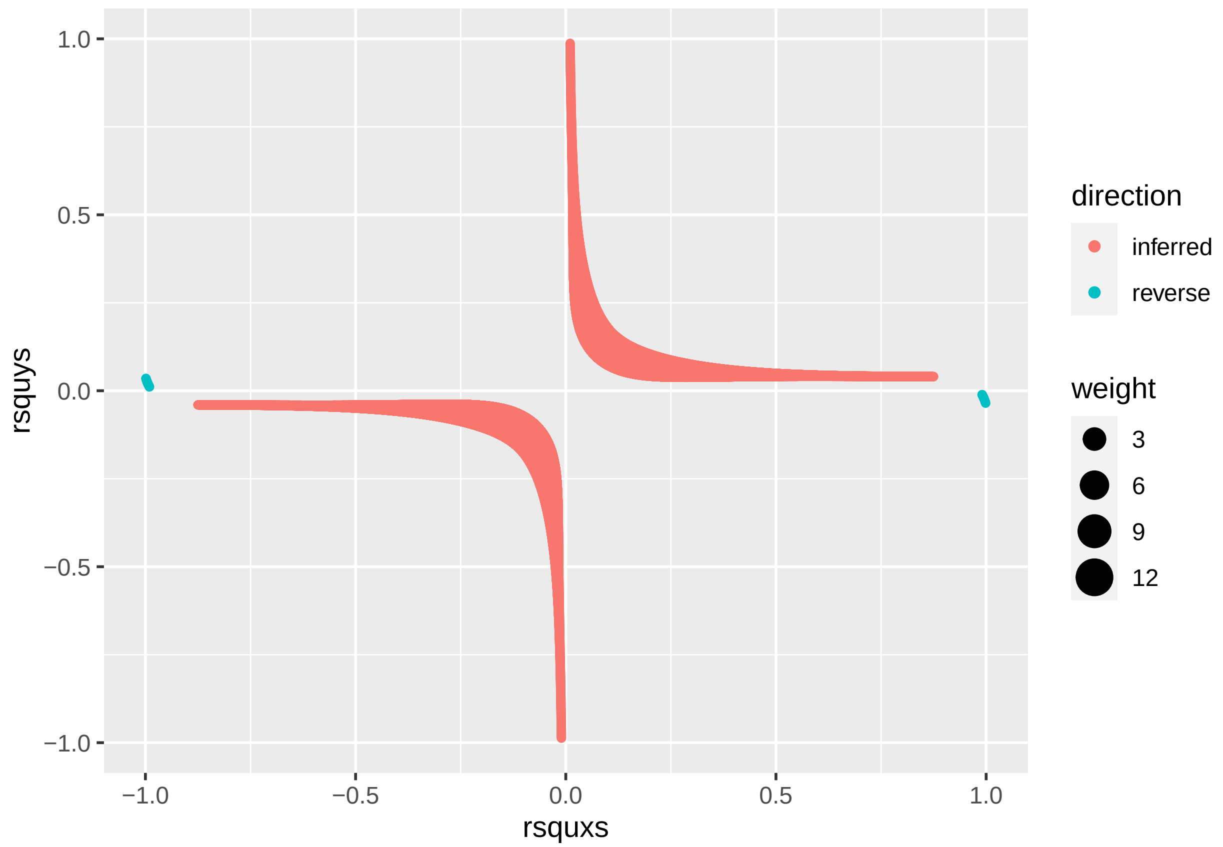

Same example but with more neutral weighting

```
r <- u_sensitivity(bxy=0.1, bxyo=0.1, bgx=0.5, vx=1, vy=1, vg=0.5, beta_a=1, beta_b=1)
r
```

```
## $result
## # A tibble: 10,044 x 25
## # Groups:   direction [2]
##      bxy   bgx    bux   buy    vg    vu      vex   vey    vx    vy  bxyo  conf
##    <dbl> <dbl>  <dbl> <dbl> <dbl> <dbl>    <dbl> <dbl> <dbl> <dbl> <dbl> <dbl>
##  1   0.1   0.5 -0.935     0   0.5     1 0         0.99     1     1   0.1     0
##  2   0.1   0.5 -0.935     0   0.5     1 0.000350  0.99     1     1   0.1     0
##  3   0.1   0.5 -0.935     0   0.5     1 0.000700  0.99     1     1   0.1     0
##  4   0.1   0.5 -0.935     0   0.5     1 0.00105   0.99     1     1   0.1     0
##  5   0.1   0.5 -0.935     0   0.5     1 0.00140   0.99     1     1   0.1     0
##  6   0.1   0.5 -0.934     0   0.5     1 0.00175   0.99     1     1   0.1     0
##  7   0.1   0.5 -0.934     0   0.5     1 0.00210   0.99     1     1   0.1     0
##  8   0.1   0.5 -0.934     0   0.5     1 0.00245   0.99     1     1   0.1     0
##  9   0.1   0.5 -0.934     0   0.5     1 0.00280   0.99     1     1   0.1     0
## 10   0.1   0.5 -0.934     0   0.5     1 0.00315   0.99     1     1   0.1     0
## # ... with 10,034 more rows, and 13 more variables: rsqgx <dbl>, rsqgy <dbl>,
## #   rsqux <dbl>, rsquy <dbl>, rsqxy <dbl>, rsqxyo <dbl>, rsqxyos <dbl>,
## #   rsquxs <dbl>, rsquys <dbl>, rsqxys <dbl>, direction <chr>, d <dbl>,
## #   weight <dbl>
##
## $prop
## [1] 0.9867418
```

```
##
## $pl
```

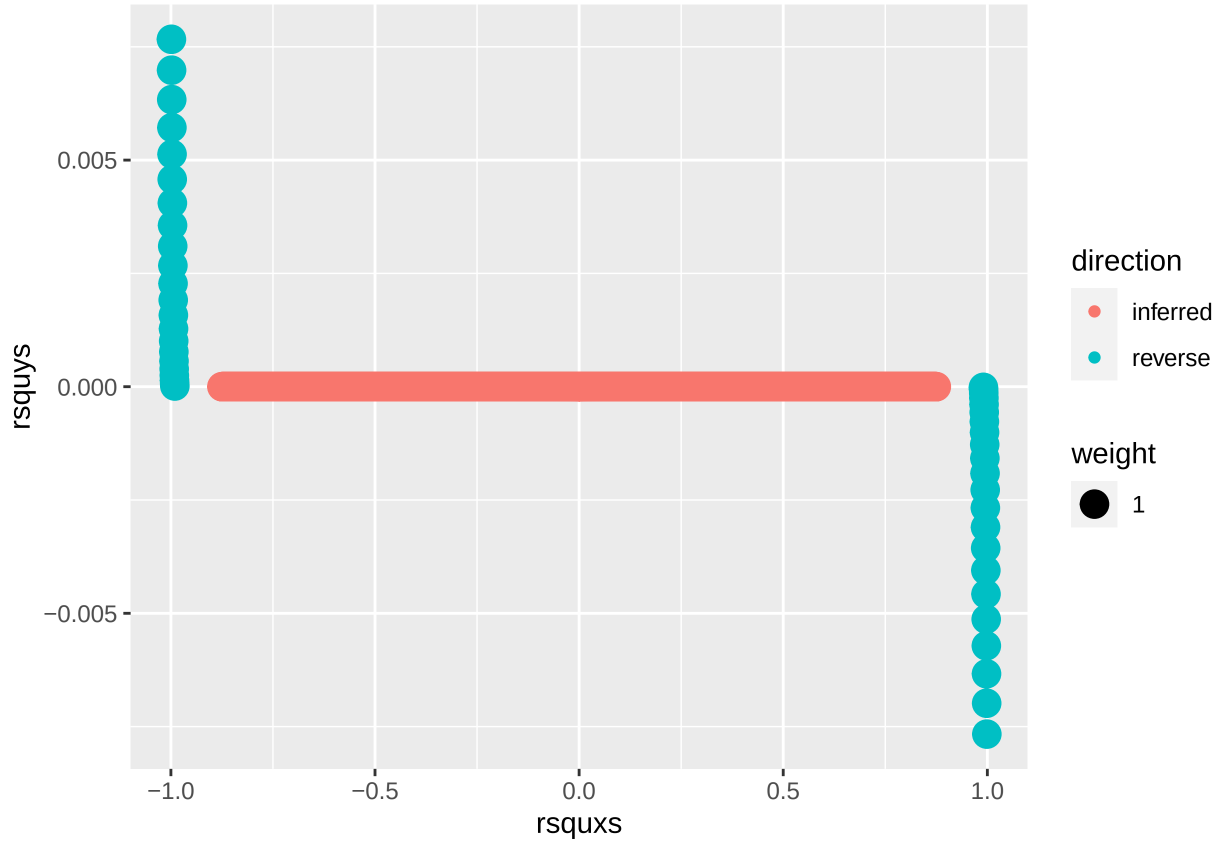

Example 2 - bxy and bxyo are very different

```
r <- u_sensitivity(bxy=0.1, bxyo=-1, vg=1, bgx=1, vx=10, vy=20, beta_a=1, beta_b=1)
r
```

```
## $result
## # A tibble: 2,212 x 25
## # Groups:   direction [2]
##      bxy   bgx   bux   buy    vg    vu     vex   vey    vx    vy  bxyo  conf
##    <dbl> <dbl> <dbl> <dbl> <dbl> <dbl>   <dbl> <dbl> <dbl> <dbl> <dbl> <dbl>
##  1   0.1     1 -3     3.67     1     1 0        8.66    10    20    -1  -1.1
##  2   0.1     1 -3.00  3.67     1     1 0.00360  8.65    10    20    -1  -1.1
##  3   0.1     1 -3.00  3.67     1     1 0.00720  8.64    10    20    -1  -1.1
##  4   0.1     1 -3.00  3.67     1     1 0.0108   8.64    10    20    -1  -1.1
##  5   0.1     1 -3.00  3.67     1     1 0.0144   8.63    10    20    -1  -1.1
##  6   0.1     1 -3.00  3.67     1     1 0.0180   8.63    10    20    -1  -1.1
##  7   0.1     1 -3.00  3.67     1     1 0.0216   8.62    10    20    -1  -1.1
##  8   0.1     1 -3.00  3.67     1     1 0.0252   8.62    10    20    -1  -1.1
##  9   0.1     1 -3.00  3.67     1     1 0.0288   8.61    10    20    -1  -1.1
## 10   0.1     1 -2.99  3.67     1     1 0.0324   8.61    10    20    -1  -1.1
## # ... with 2,202 more rows, and 13 more variables: rsqgx <dbl>, rsqgy <dbl>,
## #   rsqux <dbl>, rsquy <dbl>, rsqxy <dbl>, rsqxyo <dbl>, rsqxyos <dbl>,
## #   rsquxs <dbl>, rsquys <dbl>, rsqxys <dbl>, direction <chr>, d <dbl>,
## #   weight <dbl>
```

```
##
## $prop
## [1] 0.9433976
##
## $pl
```

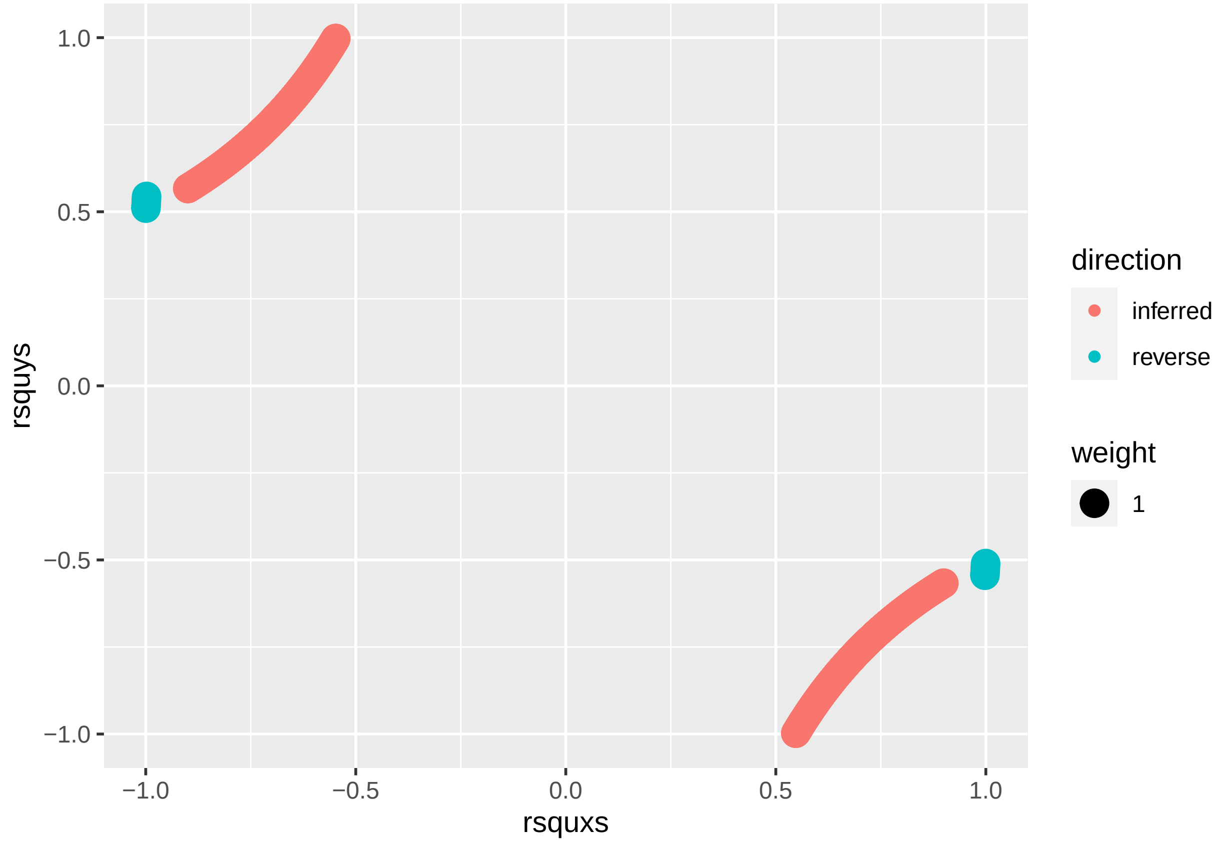

Explore general performance

```
param <- expand.grid(
    bxy = c(0.1, 0.05),
    bxyo = seq(-1, 1, by=0.05),
    vg=0.5,
    bgx = 0.01,
    vx = 4,
    vy = c(8,16),
    plot=FALSE,
    beta_a=1,
    beta_b=1
)
param$prop <- sapply(1:nrow(param), function(i) do.call(u_sensitivity, param[i,])$prop)
```

```
ggplot(param %>% filter(abs(bxyo / bxy) <=10), aes(bxyo / bxy, prop)) +
    geom_point(aes(colour=as.factor(vy))) +
    geom_line(aes(colour=as.factor(vy))) +
    ylim(c(0,1)) +
    facet_grid(. ~ bxy, labeller=label_both) +
    labs(y="Probability of correct direction", colour="Variance of Y", x=TeX(r'($\beta_{OLS}/\beta_{xy}$
```

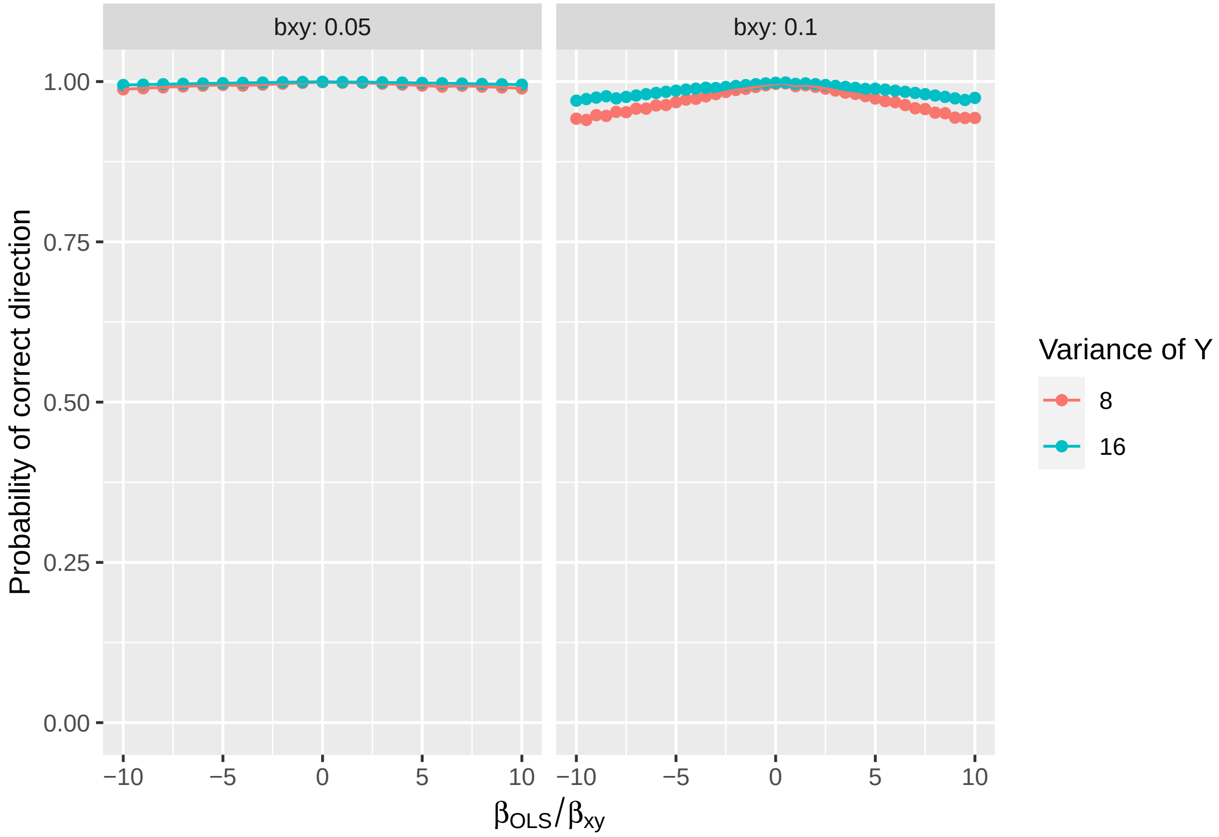

For a particular analysis, the observational association needs to be substantially larger than the causal effect in order for there to be some chance of unmeasured confounding inferring the wrong causal direction.

Empirical analysis

BMI on SBP

```
library(TwoSampleMR)
a <- extract_instruments("ieu-a-2")
b <- extract_outcome_data(a$SNP, "ukb-b-19953") %>% convert_outcome_to_exposure()
```

```
## Extracting data for 79 SNP(s) from 1 GWAS(s)
```

```
c <- extract_outcome_data(a$SNP, "ukb-b-20175")
```

```
## Extracting data for 79 SNP(s) from 1 GWAS(s)
```

```
d <- harmonise_data(b,c)
```

```
## Harmonising Body mass index (BMI) || id:ukb-b-19953 (ukb-b-19953) and Systolic blood pressure, automa
```

```
d <- add_metadata(d)
d <- add_rsq(d)

bgx <- sqrt(sum(d$rsq.exposure))
bxy <- mr(d, method="mr_ivw")$b
```

```
## Analysing 'ukb-b-19953' on 'ukb-b-20175'
```

```r
# From https://www.ncbi.nlm.nih.gov/pmc/articles/PMC6324286/
ols <- (0.8 + 1.7)/2 / 20.4 * 3.5
u_sensitivity(bxy=bxy, bxyo=ols, bgx=sqrt(sum(d$rsq.exposure)), vx=1, vy=1, vg=1, vu=1, beta_a=1, beta_
```

```
## $result
## # A tibble: 8,812 x 25
## # Groups:   direction [2]
##       bxy   bgx    bux    buy    vg    vu      vex   vey    vx    vy  bxyo  conf
##     <dbl> <dbl>  <dbl>  <dbl> <dbl> <dbl>    <dbl> <dbl> <dbl> <dbl> <dbl> <dbl>
##  1 0.0942 0.134 -0.991 -0.121     1     1 1.11e-16 0.954     1     1 0.214 0.120
##  2 0.0942 0.134 -0.991 -0.121     1     1 3.93e- 4 0.954     1     1 0.214 0.120
##  3 0.0942 0.134 -0.991 -0.121     1     1 7.86e- 4 0.954     1     1 0.214 0.120
##  4 0.0942 0.134 -0.990 -0.121     1     1 1.18e- 3 0.954     1     1 0.214 0.120
##  5 0.0942 0.134 -0.990 -0.121     1     1 1.57e- 3 0.954     1     1 0.214 0.120
##  6 0.0942 0.134 -0.990 -0.121     1     1 1.96e- 3 0.954     1     1 0.214 0.120
##  7 0.0942 0.134 -0.990 -0.121     1     1 2.36e- 3 0.954     1     1 0.214 0.120
##  8 0.0942 0.134 -0.990 -0.122     1     1 2.75e- 3 0.954     1     1 0.214 0.120
##  9 0.0942 0.134 -0.989 -0.122     1     1 3.14e- 3 0.954     1     1 0.214 0.120
## 10 0.0942 0.134 -0.989 -0.122     1     1 3.53e- 3 0.954     1     1 0.214 0.120
## # ... with 8,802 more rows, and 13 more variables: rsqgx <dbl>, rsqgy <dbl>,
## #   rsqux <dbl>, rsquy <dbl>, rsqxy <dbl>, rsqxyo <dbl>, rsqxyos <dbl>,
## #   rsquxs <dbl>, rsquys <dbl>, rsqxys <dbl>, direction <chr>, d <dbl>,
## #   weight <dbl>
##
## $prop
## [1] 0.9825434
##
## $pl
```

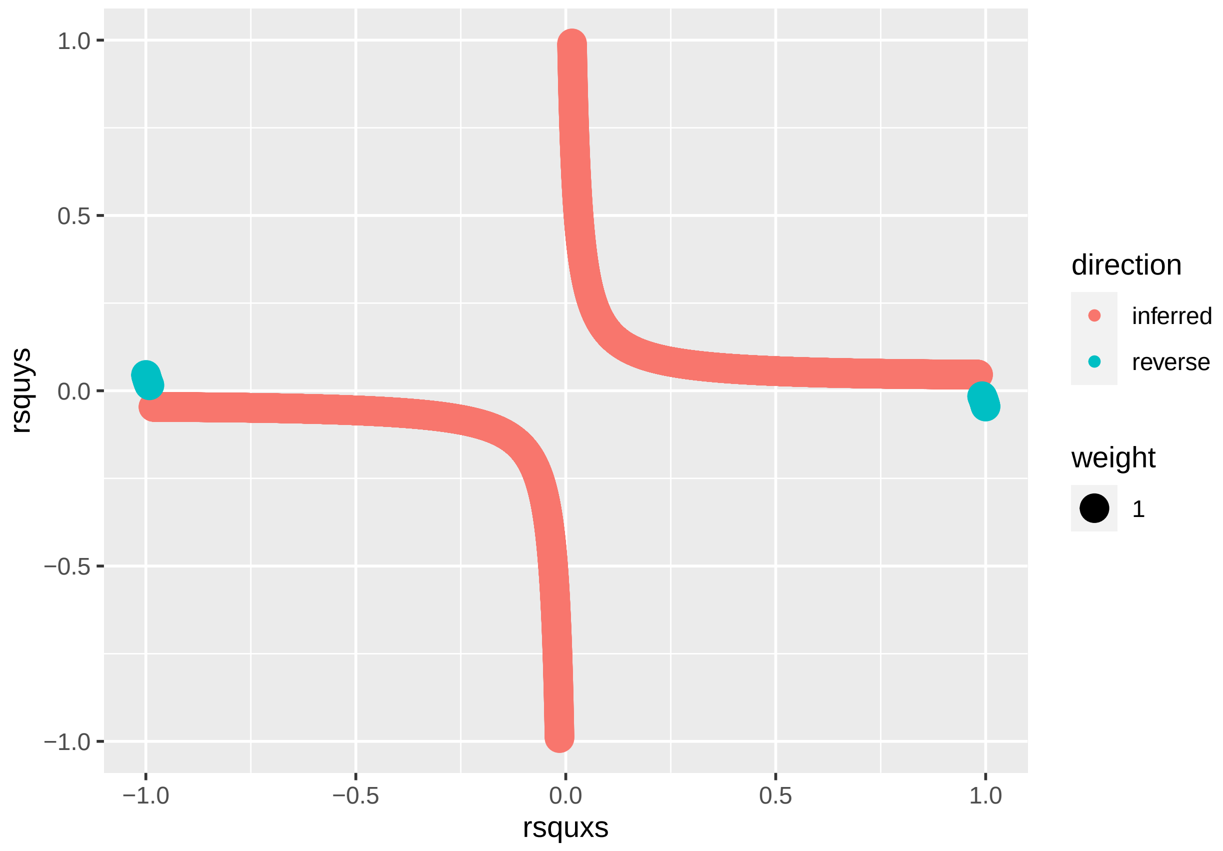
1.0
0.5
0.0
−0.5
−1.0
rsquys
−1.0
−0.5
0.0
0.5
1.0
rsquxs
direction
inferred
reverse
weight
1
